# Implications of PPPDE1 expression in the distribution of plakoglobin and β-catenin in pancreatic ductal adenocarcinoma

**DOI:** 10.3892/ol.2014.2279

**Published:** 2014-06-24

**Authors:** YU-HUAN KANG, CONG-CONG SHEN, YU-QIN YAO, LIN YU, XIN-YI CUI, YI HE, JIN-LIANG YANG, LAN-TU GOU

**Affiliations:** 1 State Key Laboratory of Biotherapy/Collaborative Innovation Center for Biotherapy, West China Hospital, West China Medical School, Sichuan University, Chengdu, Sichuan 610041, P.R. China; 2 Department of Medical Oncology, The Fifth People’s Hospital of Chengdu, Chengdu, Sichuan 611130, P.R. China

**Keywords:** PPPDE1, β-catenin, pancreatic ductal adenocarcinoma, immunohistochemistry, plakoglobin

## Abstract

Human PPPDE peptidase domain-containing protein 1 (PPPDE1) is a recently identified protein; however, its exact functions remain unclear. In our previous study, the PPPDE1 protein was found to be decreased in certain cancer tissues. In the present study, a total of 96 pancreatic ductal carcinoma tissue samples and 31 normal tissues samples were assessed to investigate the distribution of plakoglobin and β-catenin under the conditions of various PPPDE1 expression levels by means of immunohistochemistry. Generally, the staining of PPPDE1 was strong in normal tissues, but weak in cancer tissues. Plakoglobin was mainly distributed along the membrane and cytoplasm border in normal cells, but was less evident in the membranes of cancer cells. In particular, a greater percentage of cells exhibited low membrane plakoglobin expression in cancer tissue with low PPPDE1 expression (PPPDE1-low cancer) compared with that in cancer tissue with high PPPDE1 expression (PPPDE1-high cancer). The distribution of β-catenin in normal tissues was similar to that of plakoglobin. However, β-catenin was peculiarly prone to invade nucleus in PPPDE1-low cancer compared with PPPDE1-high cancer. Our data suggested potential links between PPPDE1 expression and the distribution of plakoglobin and β-catenin in pancreatic ductal adenocarcinoma, providing insights into the role of PPPDE1 in the progression of pancreatic cancer.

## Introduction

Human PPPDE peptidase domain-containing protein 1 (PPPDE1), also known as PNAS-4, is a recently identified protein through large-scale genome sequencing (1). PPPDE1 consists of 194 amino acids and contains a highly conserved DUF862 peptidase domain that is found in plants and animals, suggesting it has fundamental roles in biological evolution (2). Bioinformatics analysis has shown that PPPDE1 is a potential factor in an ubiquitin-related signaling system (3). Our previous study showed that overexpression of PPPDE1 could induce apoptosis of human lung adenocarcinoma A549 cells (4). In addition, mRNA microinjection of PPPDE1 in zebrafish and Xenopus embryos resulted in developmental defects, indicating essential roles of PPPDE1 in embryonic development (5,6).

Previously, we demonstrated that PPPDE1 was mainly located at the Golgi apparatus in the cytoplasm and presented a wide distribution in the majority of tissues, including brain, lung, kidney, liver, colon, prostate, cervix, ovary, breast and muscle tissue (7). We further found that PPPDE1 exhibited decreased expression of varying degrees in certain tumors, such as pancreatic and skin cancer, which suggested an involvement of PPPDE1 in these types of cancer (7). Pancreatic cancer is a common carcinoma with the highest mortality rate, and it is necessary to reveal its molecular mechanisms in carcinogenesis (8). Numerous studies have shown that WNT/β-catenin signaling is important in the progression of pancreatic cancer (9,10). In this process, translocation of β-catenin into the nucleus is regarded as an essential prerequisite for the transcription of a series of oncogenes including c-myc, cyclin D1 and MMP-7 (11,12). As a member of the same family as β-catenin, plakoglobin also possesses the ability to regulate cancer progression, although its roles remain controversial (13,14).

In our primary investigations, we found that PPPDE1 could affect the stability of desmosomes. Therefore, we aimed to identify the distribution of plakoglobin and β-catenin, the two important components of desmosomes, under the conditions of various PPPDE1 expression levels in pancreatic ductal adenocarcinoma. By means of analysis based on a tissue library containing 127 samples, the present study aimed to enhance the understanding of the roles of PPPDE1 and its implications in progression of pancreatic cancer.

## Materials and methods

### Antibodies

The rabbit polyclonal anti-human antibody against PPPDE1 was purchased from Proteintech (20517-1-AP; Chicago, IL, USA), while polyclonal rabbit anti-human antibodies against plakoglobin (sc-7900) and β-catenin (sc-7199) were purchased from Santa Cruz Biotechnology, Inc. (Santa Cruz, CA, USA). The polyclonal goat anti-rabbit secondary antibody conjugated with horseradish peroxidase immunoglobulin G (sc-2030) was also obtained from Santa Cruz Biotechnology, Inc. These antibodies were used according to their respective manufacturer’s instructions.

### Immunohistochemistry

The clinical specimens, including pancreatic ductal adenocarcinoma and paired normal tissues, were obtained from 96 pancreatic ductal adenocarcinoma patients (age range, 31–74 years) at the West China Hospital of Sichuan University (Chengdu, China). The study was approved by the institutional ethics committee of Sichuan University (Chengdu, China). All patient provided written informed consent to participate in the study. The sections were stained using an avidin-biotin-peroxidase complex (ABC) method and visualized with diaminobenzidine (DAB; Beyotime, Haimen, China), according to the manufacturer’s instructions. Briefly, the sections were deparaffinized in xylene and rehydrated through graded alcohol rinses. Antigen unmasking was performed by immersing slides into boiling TE-EDTA (pH 9.0) buffer and then maintaining them at boiling temperature for 15 min. After cooling, the sections were incubated in 3% H_2_O_2_ for 10 min to quench endogenous peroxidase activity. Subsequently, the sections were blocked in 5% normal goat serum for 1 h at room temperature, and incubated with primary antibodies for 3 h at room temperature. Subsequently, the sections were washed with PBS and incubated with biotinylated secondary antibodies for 30 min at room temperature. Finally, the sections were treated with ABC reagents and developed with DAB staining.

### Image analysis

Sections were counterstained with hematoxylin and imaged by using a high resolution digital microscope (Leica DM2500; Leica Microsystems GmbH, Wetzlar, Germany). The staining of sections was calculated by Leica Application Suit software (Leica Microsystems GmbH). Briefly, the values of staining intensity were measured and subjected to calibration. The staining was divided into four scales according to the size of intensity values (0–5%, negative; 6–30%, mild; 31–70%, moderate; and 71–100%, strong). The percentage of positively stained cells represents the ratio of stained cancer cells/total cancer cells. For section analysis, total staining was scored as the product of the staining intensity score (negative, 0; mild, 1; moderate, 2; and strong, 3), and the score for the percentage of positively stained cells were recorded according to an ordered categorical scale (0–5%, 0; 6–30%, 1; 31–70%, 2; and 71–100%, 3), resulting in a scale of 0–9 (15). Sections were assessed separately by two experienced pathologists. The discrepancy between the two pathologists was resolved by careful evaluation and discussion until consensus was reached.

## Results

### PPPDE1 expression analysis

**Figure 1:**
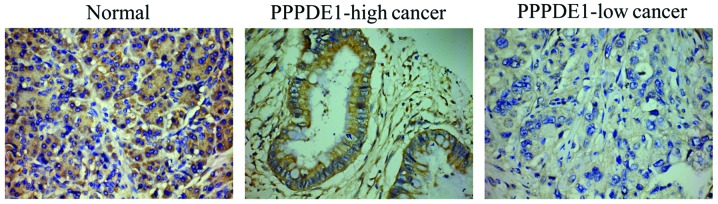
Expression analysis of PPPDE1 in pancreatic ductal adenocarcinoma and normal tissues. The expression of PPPDE1 was markedly decreased in cancer tissues compared with that in normal tissues, showing the lowest expression in poorly differentiated pancreatic cancer. PPPDE1, PPPDE peptidase domain-containing protein 1; PPPDE1-high cancer, cancer tissue with a PPPDE1 staining score of >3; PPPDE1-low cancer, cancer tissue with a PPPDE1 staining score of ≤3 (stain, diaminobenzidine; magnification, ×200).

A total of 96 pancreatic ductal carcinoma tissue samples and 31 normal pancreatic tissue samples were collected and examined to assess the expression levels of PPPDE1 by means of immunohistochemistry. Generally, strong PPPDE1 staining was evident in the normal tissues, whereas weak PPPDE1 staining was observed in the cancer tissues (Fig. 1). PPPDE1 was mainly located in the cytoplasm of the normal and cancer tissues, with almost no staining in the plasma membrane and the nucleus (Fig. 1). Notably, low PPPDE1 staining score was more evident in the poorly differentiated cancer tissues (Table I). These results confirmed that PPPDE1 presented decreased expression in pancreatic ductal adenocarcinoma, showing the lowest expression in poorly differentiated cancer tissues.

### Plakoglobin distribution in association with PPPDE1 expression

**Figure 2:**
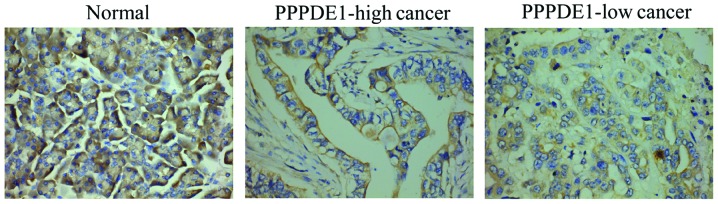
Expression analysis of plakoglobin in pancreatic ductal adenocarcinoma and normal tissues. Plakoglobin expression was highest in normal tissues compared with that in cancer tissues, and was mainly distributed in the membrane and cytoplasm border of cells. A greater proportion of cells exhibited low membrane plakoglobin expression in PPPDE1-low cancer compared with that in PPPDE1-high cancer. However, cytoplasmic staining of plakoglobin remained evident in PPPDE1-low cancer, although its total abundance was markedly decreased compared with that of normal cells. PPPDE1, PPPDE peptidase domain-containing protein 1; PPPDE1-high cancer, cancer tissue with a PPPDE1 staining score of >3; PPPDE1-low cancer, cancer tissue with a PPPDE1 staining score of ≤3 (stain, diaminobenzidine; magnification, ×200).

The tissue samples that were analyzed to assess PPPDE1 expression were also subjected to examination of plakoglobin expression, by means of immunohistochemistry. Generally, plakoglobin expression was decreased in the cancer tissues compared with that in the normal tissues (Fig. 2). The cellular location of plakoglobin distribution was then analyzed, revealing that plakoglobin was mainly distributed in the membrane and cytoplasm border of normal cells, but was less evident in the membrane of cancer cells (Fig. 2).

In order to analyze the implications of PPPDE1 expression in pancreatic ductal carcinoma, the cancer tissues were further classified into two groups, those with high (staining score, >3) levels of PPPDE1 expression (PPPDE1-high cancer) and those with low (staining score, ≤3) PPPDE1 expression levels (PPPDE1-low cancer) (Table I). A greater percentage of cells exhibited low membrane plakoglobin expression (<30%) in PPPDE1-low cancer compared with that in PPPDE1-high cancer (Table I and Fig. 2). However, cytoplasmic staining of plakoglobin remained evident in PPPDE1-low cancer, although its total abundance was markedly decreased compared with that of normal cells (Fig. 2). These results demonstrated that the membrane expression of plakoglobin shows an identical change to PPPDE1 levels in pancreatic ductal carcinoma.

### β-catenin distribution in association with PPPDE1 expression

**Figure 3:**
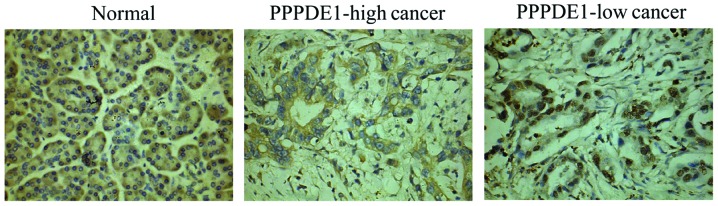
Expression analysis of β-catenin in pancreatic ductal adenocarcinoma and normal tissues. β-catenin was mainly distributed along the cytoplasm border in normal tissues. In PPPDE1-low cancer, β-catenin was particularly prone to appear in the nucleus. However, β-catenin was rarely found to invade the nucleus in PPPDE1-high cancer. PPPDE1, PPPDE peptidase domain-containing protein 1; PPPDE1-high cancer, cancer tissue with a PPPDE1 staining score of >3; PPPDE1-low cancer, cancer tissue with a PPPDE1 staining score of ≤3 (stain, diaminobenzidine; magnification, ×200).

The distribution of β-catenin in association with PPPDE1 expression in pancreatic ductal carcinoma was also investigated. Immunohistochemical analysis revealed that β-catenin was mainly distributed along the cytoplasm border in normal cells. However, β-catenin staining was most frequently observed in the cytoplasm in cancer cells. In certain representative cancer samples (Table I), β-catenin expression was identified in nucleus (Fig. 3). High nuclear β-catenin expression (>30%) was more frequently observed in PPPDE1-low cancer compared with that of PPPDE1-high cancer (Table I and Fig. 3). These results demonstrated β-catenin translocation into the nucleus was more likely to occur under the conditions of low PPPDE1 expression in pancreatic ductal carcinoma.

## Discussion

As a recently identified protein, the exact functions of PPPDE1 remain unclear. Our previous study found that PPPDE1 was located at the Golgi apparatus of HeLa cells, suggesting that it may be involved in post-translational modification in the process of protein synthesis (7). Amino acid analysis has demonstrated that PPPDE1 possesses high sequence homology to DESI-1, a deSUMOylase involving in protein modification (16). As a reverse process of SUMO, deSUMOylation mainly participates in protein stability, translocation and expression (16,17). The substrate of DESI-1 has been identified to be BZEL, but the targets of PPPDE1 remain unknown. We previously identified that PPPDE1 upregulation could lead to the weak stability of vimentin, suggesting that PPPDE1 may be involved in cytoskeleton metabolism (18). Plakoglobin and β-catenin are both important cytoskeleton-related proteins, and have been demonstrated to play important roles in cancer progression (19). In the present study, PPPDE1-low pancreatic cancer was observed to present characteristic distribution of the two cytoskeleton proteins, loss of membrane plakoglobin and translocation of β-catenin into nucleus. These findings were consistent with the high malignancy of poorly differentiated pancreatic cancer, in which PPPDE1 expression was greatly decreased. Therefore, our data provided insights into the role of PPPDE1 in the progression of pancreatic cancer.

Plakoglobin is a component of adherens junctions and desmosomes, and plays a pivotal role in the regulation of cell-cell adhesion. Previous studies have identified conflicting results regarding the function of plakoglobin in cancer progression (13,14). Numerous studies have demonstrated that plakoglobin is able to regulate the invasive properties of cancer cells (20–22). Several types of cancer, such as breast, prostate, lung, bladder, skin, thyroid, oral and pharyngeal cancer, have been found to exhibit decreased plakoglobin expression and increased the likelihood of metastasis and/or a poor prognosis (19–21).

By contrast, certain studies have demonstrated that plakoglobin exerts oncogenic activity through the triggering Tcf/Lef transcription signal, presenting similar activity to that of β-catenin (13,14,23). Notably, plakoglobin is primarily localized at the plasma membrane, with some perinuclear distribution in the cytoplasm (14). These contradictions regarding the roles of plakoglobin may be due to its differential subcellular localization. In cancer cells, the desmosomes are often destructed, resulting in the translocation of membrane plakoglobin into the cytoplasm (19). The decreased membrane plakoglobin can result in the loss of cell-cell contact and promote cell migration; however, the cytoplasm plakoglobin can liberate β-catenin from the constrained state in the cytoplasm and lead to translocation of excess β-catenin into the nucleus. Therefore, plakoglobin displays oncogenic activity through its cytoplasm accumulation to regulate the β-catenin signal, although membrane plakoglobin has been identified to be greatly decreased in cancer cells (13).

Although β-catenin is a cytoskeleton-related protein, it has been a focus of research mainly due to the WNT/β-catenin pathway, an important signaling pathway involved in development and carcinogenesis (11,24). Previous studies regarding the regulation of β-catenin translocation into the nucleus have revealed interactions between plakoglobin and β-catenin (25,26). Due to the high sequence homology between plakoglobin and β-catenin, both proteins can bind proteins such as E-cadherin, Axin and APC (13,14,25,26). This competitive binding causes the liberation of β-catenin from the Axin/APC complex and leads to β-catenin accumulation in cytoplasm. Consequently, the excess cytoplasmic β-catenin is able to translocate into the nucleus and subsequently interact with the Tcf/Lef transcription factors, which triggers the expression of a series of oncogenes, such as c-myc, cyclin D1 and MMP-7. In the present study, it was identified that the cellular distribution of plakoglobin and β-catenin may be regulated by PPPDE1 expression in pancreatic ductal carcinoma. Although the mechanisms whereby PPPDE1 expression is decreased in pancreatic ductal carcinoma remain unclear, the intracellular distributions of plakoglobin and β-catenin in association with PPPDE1 expression suggested there may be certain connections among these proteins.

In summary, the findings of the present study suggested that there may be associations between PPPDE1 expression and the distribution of plakoglobin and β-catenin expression in pancreatic ductal adenocarcinoma. The characteristic cellular distributions of plakoglobin and β-catenin in pancreatic cancer have provided insights into the role of PPPDE1 in pancreatic cancer progression. The molecular mechanisms underlying the characteristic distributions in association with PPPDE1 expression require further investigation on the interacting proteins of PPPDE1 with regard to their interactions in the future.

## Figures and Tables

**Table I:** Subcellular distributions of plakoglobin and β-catenin under different PPPDE1 expression levels.

	Pancreatic ductal adenocarcinoma	
	
	Low PPPDE1	High PPPDE1
Staining score	≤3	>3
Sample (n)	44	52
Poor differentiation	73% (32/44)	23% (12/52)
Membrane plakoglobin (<30%)	57% (25/44)	27% (14/52)
Nucleus β-catenin (>30%)	64% (28/44)	19% (10/52)

PPPDE1, PPPDE peptidase domain-containing protein 1.
